# Cephalometric Pattern and Nasal Patency in Children with Primary Snoring: The Evidence of a Direct Correlation

**DOI:** 10.1371/journal.pone.0111675

**Published:** 2014-10-31

**Authors:** Anna Maria Zicari, Marzia Duse, Francesca Occasi, Valeria Luzzi, Emanuela Ortolani, Flaminia Bardanzellu, Serena Bertin, Antonella Polimeni

**Affiliations:** 1 Department of Pediatrics, “Sapienza” University of Rome, Rome, Italy; 2 Department of Oral and Maxillo-Facial Sciences, “Sapienza” University of Rome, Rome, Italy; 3 George Eastman “Odontoiatric hospital”, Rome, Italy; 4 Department of pediatric otorhinolaryngology, “Sapienza” University of Rome, Rome, Italy; Fondazione IRCCS Ca' Granda Ospedale Maggiore Policlinico, Università degli Studi di Milano, Italy

## Abstract

**Introduction:**

Sleep disordered breathing (SDB) might affect craniofacial growth and children with obstructive sleep apnea syndrome present an increase in total and lower anterior heights of the face and a more anterior and inferior position of the hyoid bone when compared to nasal breathers.

**Objective:**

To investigate the correlation between rhinomanometric and cephalometric parameters in children with primary snoring (PS), without apnea or gas exchange abnormalities.

**Materials and Methods:**

Thirty children with habitual snoring (16 females and 14 males) aged 4–8 years (mean age 6.85±1.51 years) were selected by a SDB validate questionnaire. All subjects underwent lateral cephalometric, panoramic radiographies.

**Results:**

In our sample 10 children (33%) had snoring 3 nights/week, 11 (37%) 4–6 nights/week and 9 (30%) every night/week. Overall 7 patients (23.3%) were affected by adenoid hypertrophy (AH), 4 (13.3%) by tonsillar hypertrophy (TH) and 13 (43.3%) by AH and TH. We found a more vertical position of the hyoid bone to the mandibular plane (H⊥VT) in patients with a higher frequency (7.3±2.7 vs 7.6±3.7 vs 10.9±2.5 in children snoring 3 nights/week, 4–6 nights/week and every night/week respectively; p = 0.032). Concerning nasal patency significant correlations were found with ANB (maxillary and jaw position with respect to the cranial base), NS∧Ar (growth predictor), sumangle, FMA (total divergence), SnaSnp∧GoMe (inferior divergence), BaN∧PtGn (facial growth pattern), Phw1_PsP (posterosuperior airway space), AHC3H (the horizontal distance between the most anterosuperior point of the hyoid bone and the third cervical vertebra).

**Conclusion:**

The present study supports the relationship between nasal obstruction and specific craniofacial characteristics in children with primary snoring and lead us to hypothesize that nasal obstruction might explain the indirect link between snoring and cephalometric alterations.

## Introduction

In the spectrum of Sleep Disordered Breathing (SDB), snoring affects 26% of school aged children and it is considered a primary symptom of upper airway obstruction ranging in severity from primary snoring (PS) (no evidence of ventilation abnormalities) to severe obstruction characterized by gas exchange abnormalities and frequent nocturnal arousals [1]. Mouth breathing as a consequence of increased nasal resistance [2] is strongly and independently associated with Habitual Snoring (HS). Although an increased nasal resistance is often found in children with chronic nasal congestion and/or enlarged tonsils and/or adenoids, mouth breathing per se may lead to an irritation of tissues promoting viral infections and hence an Enhanced growth of lymphatic tissue within the upper airway [3].

The impact of the mode of breathing on craniofacial and dentofacial growth has been the object of many controversies over years. The harmonious development of craniofacial structures seems to be negatively influenced by the instauration of nasal breathing [4]. Rhinitis and adenoid hypertrophy (AH), increasing the air intake through the mouth, can be considered as indirect causes of a functional change of the neuro-muscular system and of the head and neck region with an altered bone and soft tissues structure development [5]. In this perspective, SDB including a broad spectrum of alterations, ranging from PS to obstructive sleep apnea syndrome (OSAS), might affect craniofacial growth and children experiencing these symptoms present an increase in total and lower anterior heights of the face and a more anterior and inferior position of the hyoid bone when compared to nasal breathers [6]. Although many authors have studied rhinomanometric and cephalometric parameters, according to our knowledge, results focusing specifically on this correlation are lacking and studies performed in children with primary snoring, without apnea or gas exchange abnormalities, are few.

## Material and Methods

Between July 2012 and December 2013, at the Allergology and Immunology service of the Pediatric Department of the hospital “Policlinico Umberto I” in Rome, 30 children with Habitual snoring (HS) (16 females and 14 males) aged 4–8 years (mean age 6.85±1.51 years) were consecutively selected by a questionnaire validated to assess pediatric SDB composed by 51 item multiple choice [7], [8]. Snoring was investigated with the question: “Does your child snore?”; responses were rated on a 4-point rating scale (0 = never, 1 = occasionally, 2 = frequently or 3 = always). The questionnaires were all completed by one investigator who interviewed the patients. When necessary, parents were asked to help the child without interfering with his/her responses. Children were considered affected by habitual snoring when the symptom was reported 3 or more nights per week since at least six months.

For each child a detailed clinical history was collected and a complete physical examination was performed. Nasal Fibroptic Endoscopy NFE was performed by an expert otorhinolaryngologist using a 2.7 mm diameter endoscope and children were considered affected by AH when adenoids occluded more than 25% of the coanal opening using Cassano et al. criteria [9]. Moreover Tonsillar hypertrophy (TH) was graded as follows: Grade 1-tonsils are in the tonsillar pillar, Grade 2-tonsils are protruding out of the tonsillar pillar, Grade 3-tonsils reaching midpoint between anterior tonsillar pillar and uvula, Grade 4-tonsils reach the uvula [10]. Atopic status was assessed by a Skin Prick Test (SPT) (Lofarma, Milan, Italy) and/or elevated specific (>0.35 kU/l) and total IgE (>100 kU/l). Panels included the following allergens: Dermatophagoides pteronissynus, Dermatophagoides farina, dog/cat dander, Olea europea, Lolium perenne, Alternaria tenuis, Parietaria officinalis, lactalbumin, ß-lactoglobulin,casein, egg white and yolk, soy, codfish. A positive SPT was defined by the presence of a heal more than 3 mm respect to the wheal size of control (saline solution) [11].

Subjects with the following characteristics were excluded from the study: overweight or obesity (BMI>85° percentile), craniofacial anomalies or a syndromic disorder genetic, chronic medical conditions, intercurrent upper respiratory tract or systemic infection within 4 weeks of recruitment, neuromuscular disorder or if they had previously undergone upper airway surgery.

The study was approved by the International Review Board of “Sapienza” University of Rome and performed with the written parental informed consent.

### Nocturnal Pulse Oximetry and Polysomnography

All children selected for HS underwent a nocturnal pulse oximetry and a polysomography. The pulse oximetry was performed according to by Brouillette et al. [12] and Nixon et al. [13] with a motion-resistant pulse oximeter set for a 2-sec averaging time for hemoglobin saturation (SpO2) (RAD 5, Masimo, Irvine, CA).

Pulse oximetry data were extracted and analyzed with Profox Oximetry Software (Profox Associates, Escondido, CA). A recording was considered sufficient when the artifact-free recording time was at least 6 hours.

According to the Brouillette definitions and criteria each oximetry was classified as positive, negative or not conclusive [14] using the following definitions and criteria: 1) A desaturation was defined as a decrease in Sao2 of 4% or more; 2) A cluster of desaturations was defined as 5 or more desaturations occurring in a 10- to 30-minute period; 3) On the oximetry trend graphs, periods of relative tachycardia, usually 10 to 25 beats per minute, at the beginning and end of nocturnal pulse oximetry, and periods of relative tachycardia and increased heart rate variability exceeding 30 minutes were regarded as wakeful time and not considered; 4) Event graphs for Sao2 were used to distinguish true desaturations from movement artifacts and low signal amplitude artifacts using a method we have termed pulse amplitude modulation range16; 5) A positive oximetry trend graph had 3 or more desaturation clusters and at least 3 desaturations to,90%19; 6) A negative oximetry trend graph had no desaturation clusters and no desaturations to,90%; and 7) An inconclusive oximetry trend graph was 1 that did not meet the criteria for positive or negative.

An overnight polysomnography (PSG) was performed after admission to the hospital on all children selected for HS to record the following parameters: six EEG channels (Fp1-A2, Fp2-A1, C3- A2, C4-A1, O1-A2, and O2-A1 electrode placement according to the international 10–20 system), left and right electrooculogram (EOG), chin electromyogram (EMG), electrocardiogram (ECG), electromyogram of left and right tibialis anterior muscles, nasal flow, thoracic and abdominal respiratory effort, body position and oxygen saturation. The obstructive apnea-hypopnea index (OAHI) was defined as the total number of apneic and hypopneic episodes per hour of sleep. Hypopnea was considered as a reduction of 50% or more in the amplitude of the airflow signal and it was only quantified if longer than two baseline breaths and associated with oxygen desaturations of at least 4% and/or arousals. A diagnosis of PS was given if OAHI was <1 and SpO2 nadir was ≥90%, while a diagnosis of OSA if OAHI was ≥1 [15]. Children with OSA were excluded from the study.

### Anterior active rhinomanometry

The same day patients underwent Anterior Active Rhinomanometry (Sibelmed Rinospir PRO 164) in accordance with the International Committee on Standardization of rhinomanometry [16]. A retest was performed in all patients. The results of rhinomanometry were considered related to nasal flows of 150 Pascal (Pa) and compared with pediatric reference values height-dependent reported in literature [17]. According to Zapletal et al. classification rhinomanometry was considered negative (no nasal obstruction) when the fraction of predicted values (p.v.) was ranged between 71% and 100% while it was considered positive when the fraction of p.v. was <70%.

### Cephalometric evaluation

**Figure 1:**
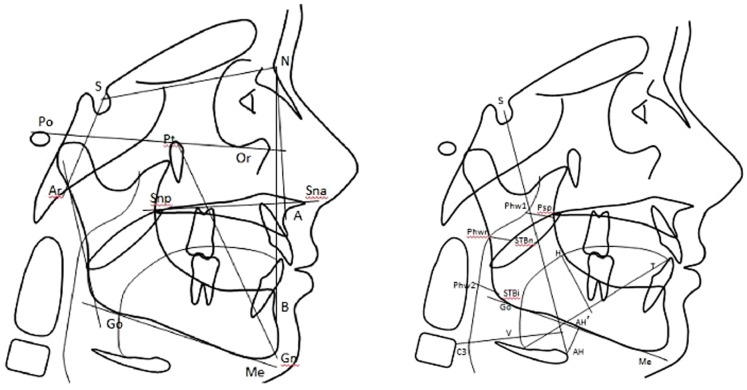
SNA (degrees), SNB (degrees), and ANB (degrees) were used to define the maxillary and jaw position with respect to the cranial base (SN) and the respective skeletal Class; NS∧Ar (degrees), SAr∧Go (degrees), ArGo∧Me (degrees) study growth direction; BaN ∧ PtGn is the upper angle, measured between total cranic base (BaN) and facial axis (Pt Gn) and defines the facial growth; Po-Or ∧GoMe or FMA (degrees) is measured between the Frankfort horizontal plane and the mandibular plane and represents the total divergence while PFH∧SnaSnp the superior divergence and SnaSnp∧GoMe, the inferior divergence. AH-C3H (mm), the horizontal distance from AH to C3; AH-AH' (mm), the vertical position of the hyoid to the mandibular plane; H VT, representing the vertical position of the tongue; Phw1-Psp (mm), posterior superior airway space; PhwN-STBn, the middle pharyngeal width; PhwI-STBi, inferior airway space.

**Figure 2:**
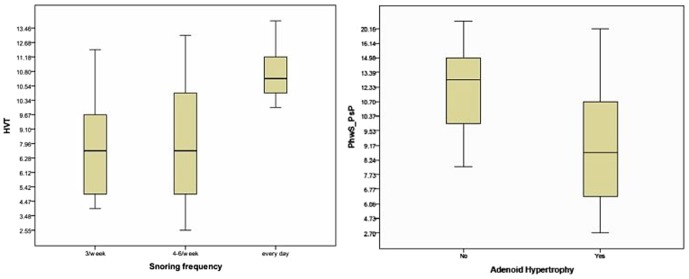
The vertical position of the tongue represented by H⊥VT in children reporting a different snoring frequency (7.3+2.7 vs 7.6+3.7 vs 10.9+2.5 in children snoring 3 nights/week, 4–6nights/week and every day respectively; p = 0.032) and the posterior superior airway space or Phw1_PsP in children with and without adenoid hypertrophy (9.3+4.5 vs 17.1+4.6; p<0.01).

All subjects underwent lateral cephalometric, panoramic radiographies and dental impression in the Department of Oral and Maxillo-Facial Sciences, “Sapienza” University of Rome. Cephalograms were obtained with the teeth in centric occlusion and with the lips in light contact. Centric occlusion was used to minimize variability in mandibular and soft tissue measurements, often associated with rest position. The lateral cephalogram was taken using natural head posture, found by having the patient look in front of him or herselves. All cephalometric landmarks were located and digitized by the same investigator using the Oris ceph software and was evaluated according to Kulnis et al. [18] as showed in Figure 1 and Figure 2. For the sagittal analysis, angles SNA (degrees), SNB (degrees), and ANB (degrees) were used to define the maxillary and jaw position with respect to the cranial base (SN) and the respective skeletal Class. For the vertical analysis we used the angle FMA (degrees), measured between the Frankfort horizontal plane and the mandibular plane, representing the total divergence, the angles PFH∧SnaSnp for the superior divergence and SnaSnp∧GoMe for the inferior divergence. Concerning growth predictors: NS∧Ar (degrees), SAr∧Go (degrees), ArGo∧Me (degrees) and respective sumangle based on Jarabak analysis (the increase sumangle correspond to a posterior rotation of the jaw and to a greater vertical growth); BaN∧PtGn, based on Ricketts analysis, is the upper angle, measured between total cranic base (BaN) and facial axis (Pt Gn) and it defines the facial growth, with an increased angle value corresponding to a prevalent horizontal growth and the reverse if reduced. The hyoid bone position was evaluated with AH-C3 H (mm), the horizontal distance from AH to C3; AH is the most anterior and superior point on the body of the hyoid bone and represents the inferior part of the tongue; C3 is the third cervical vertebra; AH-AH' (mm) is the vertical position of the hyoid to the mandibular plane. H⊥VT represents the vertical position of the tongue; VT is the distance from the intersection of the epiglottis and the base of the tongue to the tip of the tongue. Soft tissue and oropharyngeal dimension were measured according to Pracharktam et al. [19]: Phw1-Psp (mm), posterosuperior airway space measured along a line parallel to B-Go; PhwN-STBn, the middle pharyngeal width measured between posterior pharyngeal wall at narrowest point and the most anterior point in the airway on the soft palate; PhwI-STBi, inferior airway space between posteroinferior pharyngeal wall and the most anterior point in the airway on the tongue.

### Statistical analysis

Statistical analyses were performed using SPSS (Statistical Package of Social Sciences, Chicago, IL, USA) software version 19. Descriptive statistics were performed expressing continuous data as means with SDs, or as medians with interquartile ranges and categorical data were expressed by frequency and percentage. Comparisons were evaluated using a t-test, a chi-square test, or a Mann-Whitney U-test while correlations between cephalometric and rhinomanometric parameters were calculated with Pearson's correlation test. A p-value less than 0.05 was considered statistically significant.

## Results

In our sample 10 children (33%) had snoring 3 nights/week, 11 (37%) 4–6 nights/week and 9 (30%) every night/week. Overall 7 patients (23.3%) were affected by AH, 4 (13.3%) by TH and 13 (43.3%) by AH and TH (Table 1). Nine children (30%) had a positive SPT: 5 to dust mites, 1 alternaria alternata, 2 parietaria officinalis, 3 olea, 5 grass mix, 1 cat dander and 1 milk. Moreover snoring frequency was comparable between females and males (p = 0.79) and between children with and without AH and TH (p = 0.27) (Table 1). Cephalometric parameters were not related with age and were similar between males and females and between atopic and not atopic children (data not shown). As shown in figure 2, comparing cephalometric parameters among children with a different snoring frequency we found a higher H⊥VT in patients with a higher frequency (7.3±2.7 vs 7.6±3.7 vs 10.9±2.5 in children snoring 3 nights/week, 4–6 nights/week and every night/week respectively; p = 0.032). Furthermore, children with AH reported a lower Phw1_PsP (9.3±4.5 vs 17.1±4.6; p<0.01; Figure 2) and PhwN_STBn (5.8±2.5 vs 9.2±1.7; p<0.03) while no difference was found for TH.

**Table 1:** Severity of snoring and nasal patency stratified according to sex, adenoid (AH), tonisllar (TH), adenoid plus tonsillar hypertrophy (AH and TH) and no adenoid or tonsillar (No AH or TH) hypertrophy.

Nasal Fibroptic Endoscopy		Nr	Snoring 3 ngt/wk	Snoring 4–6 ngt/wk	Snoring every ngt/wk	Nasal Patency
**No AH or TH**		6 (20%)	3 (50%)	2 (33%)	1 (17%)	54±25.84
**AH**		7(23.3%)	2 (28.6%)	1 (14.3%)	4 (57.1%)	42.14±24.98
**TH**		4(13.3%)	1 (25%)	3 (75%)	0	55.75±26.64
**AH and TH**		13(43.3%)	4 (30.8%)	5 (38.5%)	4 (30.8%)	48.34±23.23
**AH**	grade 2	6 (30%)	2 (33.3%)	2 (33.3%)	2 (33.3%)	34.08±25.01
	grade 3	4 (20%)	3 (75%)	0	1 (25%)	45.87±20.42
	grade 4	10 (50%)	1 (10%)	4 (40%)	5 (50%)	53.55±22.6
**TH**	grade 2	9 (52.9%)	4 (44.4%)	5 (55.6%)	0	30.87±10.29
	grade 3	6 (35.3%)	1 (16.7%)	3 (50%)	2 (33.3%)	46.66±10.88
	grade 4	2 (11.8%)	0	0	2 (100%)	63.75±10.25
**Male**		14 (47%)	4 (28.6%)	5 (35.7%)	5 (35.7%)	47.11±18.75
**Female**		16 (53%)	6 (37.5%)	6 (37.5)%	4 (25%)	53.42±29.40

**Figure 3:**
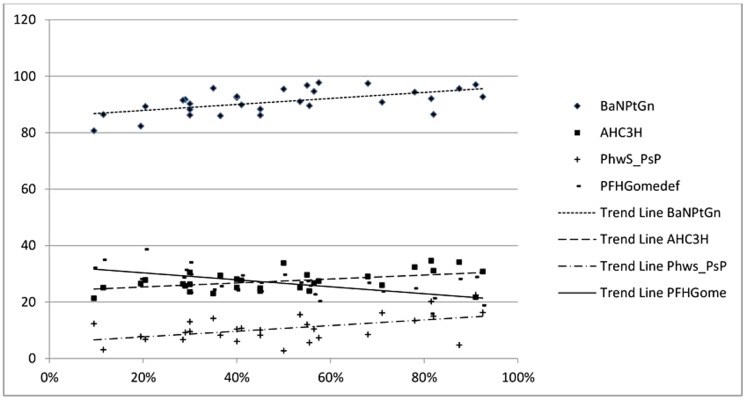
Correlations between nasal patency as fraction of predicted values of nasal flows, BaN∧PtGn, the upper angle measured between total cranic base (BaN) and facial axis (Pt Gn) (r 0.570; p<0.001) and AH-C3 H, the horizontal distance from AH to C3 (r 0.46; p = 0.009).

Concerning nasal patency, the fraction of predicted values of nasal flows at the rhinomanometric evaluation did not vary for sex and AH and/or TH (p = 0.52; p>0.05) even though significant correlations were found with ANB, NS∧Ar, sumangle, FMA, SnaSnp∧GoMe, BaN∧PtGn, Phw1_PsP, AHC3H, as shown in Figure 3 and reported in Table 2.

**Table 2:** Cephalometric parameters in our sample of children with primary snoring and their correlation with nasal patency as fraction of predicted values of nasal flows expressed by Pearson's correlation coefficient.

Parmeters	Mean values	SD	Correlation coefficient (r)	Correlation significance (p)
**SNA (°)**	82.00	4.27	0.05	0.78
**SNB (°)**	77.41	3.45	−0.25	0.17
**ANB (°)**	4.58	2.80	0.38	0.03*
**NS∧Ar (°)**	122.34	6.43	−0.47	0.008*
**Sar∧Go (°)**	145.04	6.88	0.09	0.61
**ArGo∧Me (°)**	128.44	5.73	−0.11	0.54
**Sumangle (°)**	395.83	5.19	−0.58	0.001*
**FMA (°)**	26.73	4.81	−0.61	<0.0001
**PFH∧SnaSnp (°)**	3.42	2.38	−0.26	0.15
**BaN∧PtGn (°)**	90.9	4.4	0.57	0.001*
**SnaSnp∧Gome (°)**	29.94	4.56	−0.52	0.003*
**PhwN_STBn (mm)**	6.8	2.6	0.06	0.75
**Phw1_PsP (mm)**	10.52	4.69	0.51	0.004*
**PhwI_STBi (mm)**	12.71	2.72	0.15	0.40
**H⊥VT (mm)**	8.5	3.3	0.14	0.435
**AH-C3 H (mm)**	27.3	3.5	0.468	0.009*
**AH-AH' (mm)**	12.3	3.3	−0.32	0.076

## Discussion

Although it is still a matter of debate whether mouth breathing is the cause or the consequence of craniofacial alteration and if the structure of the facial skeleton directly influences the development of the size of adenoids, our aim was to assess the direct correlation between nasal patency and craniofacial characteristics to better define the relationship between snoring and cephalometric patterns.

Craniofacial characteristics of children with habitual snoring have been extensively studied during the last decades and they still represent the object of many controversies. Although it is not possible to identify a direct relationship between the cause of respiratory obstruction and its effect on craniofacial growth [20], [21], [22], mouth breathing may related to an alteration of the position of the orofacial muscles and of the mandible influencing mastication, deglutition and phonation, leading to occlusal and skeletal alterations [23]. Since 1970's many authors reported, in mouth breathing children, an increased total and lower anterior facial height and a dolicofacial pattern rather than a mesofacial pattern [24], [25], [26]. In this perspective the long face pattern and the vertical growth of the face seems to be closely related to mandibular rotation in these patients [27]. In our sample of children with PS nasal patency was related with BaN PtGn, NS∧Ar and sumangle suggesting that a posterior rotation of the jaw and a greater vertical growth may be associated with nasal obstruction. Moreover the importance of the maxillary and the jaw position is confirmed by the correlation between FMA, SnaSnpGome and nasal patency.

In 1980 Sorensen et al. [28] reported a correlation between Nasal resistance and the distance between the most anterior part of the adenoidal mass to the posterior wall of the upper part of the nasopharynx. This distance was related also to the symptom snoring. Recently Akpinar et al. [29] identified in children with OSA and HS an increased mandibular plane and hyoid distance, soft palate length and a decreased posterior airway space as the determinant characteristics. In line with these findings we found a correlation between the horizontal distance C3-hyoid bone (AHC3H) and nasal obstruction and a vertical position of the tongue (H⊥VT) in children snoring more frequently supporting the importance of hyoid bone position. Furthermore, concerning posterior airway space, we found a reduction of postero superior pharyngeal lumen (Phw1-Psp) in patients with a lower nasal patency even though the difference of nasal flow between children with and without AH did not reach the significance level.

The most important limitation of our study may be represented by the number of children studied and the small samples after stratification according to adenoid/tonsillar hypertrophy and the severity of snoring. Further studies on larger number of subjects are warranted to better quantify the importance of AH on cephalometric pattern and nasal patency.

The cephalometric influence of nasorespiratory obstruction and its effects on facial growth continues to be debated after almost a century of controversy and the role of nasal obstruction is fueled by strong convictions, weak evidence, and the prevailing uncertainty of cause and effect relationships that exist. The sequence of events may lead from nasal obstruction through lip apart posture and mouthbreathing to modification of facial growth [30]. Recently Sin S et al. [31] have shown that obese children with OSAS have an increase in nasal resistance that preloads the nasopharynx and oropharynx, and that this resistance correlates with the severity of the disorder. These findings may explain the low response to adenotonsillectomy in these subjects suggesting that the relationship between nasal resistance and OSAS is not well defined and that other factors, not ameliorated by adenotonsillectomy, such as low upper airway muscle tone, increased parapharyngeal fat and anatomical abnormalities of the nasal passages may contribute to SDB in children. Chronic nasal obstruction can occur due to a variety of causes including deviated nasal septum, hypertrophied tissue at the maxillary tuberosity, allergic rhinitis [32] and enlargement of adenoids/tonsillar tissue should not be regarded as the only protagonist when assessing the cause and effect relationships between nasorespiratory obstruction and SDB in children. In these purpose, cephalometric characteristics might directly influence nasal patency and may not be regarded merely as the effect or the cause of AH and, hence, of a reduction of nasal patency.

In conclusion, the results of the present study support the relationship between nasal obstruction and specific craniofacial characteristics of children with HS and lead us to hypothesize that nasal obstruction might explain the indirect link between snoring and cephalometric alterations. Our findings underline the role of the assessment of nasal obstruction in children affected by PS with objective methods such as rhinomanometry and highlight the importance of an accurate orthodontic evaluation.
